# What can we talk about, in which language, in what way and with whom? Sami patients' experiences of language choice and cultural norms in mental health treatment

**DOI:** 10.3402/ijch.v74.26952

**Published:** 2015-05-13

**Authors:** Inger Dagsvold, Snefrid Møllersen, Vigdis Stordahl

**Affiliations:** 1 Sámi Norwegian National Advisory Unit on Mental Health and Substance Use, Finnmark Hospital Trust, Hammerfest, Norway; 2 Centre for Sami Health Research, The Faculty of Health Science, UiT The Arctic University of Norway, Tromsø, Norway

**Keywords:** Norway, Sami, mental health, qualitative, experiences, language, culture, cultural norms

## Abstract

**Background:**

The Sami in Norway have a legal right to receive health services adapted to Sami language and culture. This calls for a study of the significance of language choice and cultural norms in Sami patients’ encounters with mental health services.

**Objectives:**

To explore the significance of language and cultural norms in communication about mental health topics experienced by Sami patients receiving mental health treatment to enhance our understanding of linguistic and cultural adaptation of health services.

**Methods:**

Data were collected through individual interviews with 4 Sami patients receiving mental health treatment in Northern Norway. A systematic text reduction and a thematic analysis were employed.

**Findings:**

Two themes were identified:

(I) Language choice is influenced by language competence, with whom one talks and what one talks about.

Bilingualism was a resource and natural part of the participants’ lives, but there were limited possibilities to speak Sami in encounters with health services. A professional working relationship was placed on an equal footing with the possibility to speak Sami.

(II) Cultural norms influence what one talks about, in what way and to whom.

However, norms could be bypassed, by talking about norm-regulated topics in Norwegian with health providers.

**Conclusion:**

Sami patients’ language choice in different communication situations is influenced by a complexity of social and cultural factors. Sami patients have varying opinions about and preferences for what they can talk about, in which language, in what way and with whom. Bilingualism and knowledge about both Sami and Norwegian culture provide latitude and enhanced possibilities for both patients and the health services. The challenge for the health services is to allow for and safeguard such individual variations within the cultural framework of the patients.

Cultural difference often serves as one among other explanations of why indigenous people have poorer health and are less satisfied with health services than majority populations, and cultural adaption of health services and cultural competence in health providers are assumed to improve health services and reduce health differences (1–5). In Norway too, cultural and linguistic differences have been an explanation of the differences found in health status between Norwegians and the indigenous people, the Sami. Thus, after the Second World War, two strategies were attempted to reduce the health gap: the welfare state model ensuring equal access to decentralized health services and the occasional recognition of the significance of Sami language and culture in health services for the Sami (6–11). Recent research indicates minor differences in health status and use of health services between Norwegians and Sami (12–16). However, Sami identity can still be a stigma and some studies indicate a correlation between discrimination and mental health problems (17). In spite of minor differences in health status^1^ and in use of health services, studies indicate that the Sami are less satisfied (18–20) and have greater communication problems (21) with the health services than Norwegians because of linguistic and cultural differences. This is the point of departure in the present study, which explores the significance of linguistic and cultural aspects in interaction and communication about mental health topics, as experienced by Sami patients.

The Sami population resides in Norway, Sweden, Finland and the Kola Peninsula in Russia and is estimated to number about 100,000.^2^ The majority of the Sami, roughly 40,000, live in Norway,^3^ maintaining their own language and culture. Historically, from the middle of the nineteenth century, the Sami in Norway experienced a 100- to 150-year-long period of harsh assimilation policy, leading to a stigmatized view of Sami identity, culture and language as inferior. The idea of belonging to an inferior race gave rise to shame and self-hatred in the population, which made a great many Sami hide their identity and speak Norwegian rather than Sami (22). The Sami-policy in Norway has gradually changed from assimilation towards cultural safeguarding and a revitalization of Sami society. Norway formally approved the Sami as an indigenous people in 1990. The prevailing understanding is that to achieve the goal of equitable health services for the Sami people, such health services should adapt to Sami language and culture (6,21,23).

Health services for the Sami are integrated in the public welfare state system in Norway. As Norwegian citizens, the Sami are legally entitled to public health services, and as an ethnic minority^4^ and indigenous population,^5^ to culturally and linguistically adapted health services. The Sami Act of 1987, § 3–5, regulates the right to use Sami to protect his or her own interests vis-à-vis local, regional or national public health and social institutions if their services cover parts of the Sami Language Administrative District. In addition, The Sami National Centre for Mental Health and Substance Abuse (SANKS) was established in 2002. SANKS is integrated into Finnmark Hospital Trust and serves the whole population in the Mid-Finnmark area, but holds a special obligation to develop mental health services for the Sami throughout Norway.^6^

The Sami in Norway speak 3 main Sami languages, following the borders in Northern, Lule and Southern Sami settlement areas, with several dialects within each language (24). The number of Sami speakers (and the number of Sami-speaking therapists^7^) in Norway is unknown. However, an estimate indicates that more than half of the Sami population in Norway is bilingual in Norwegian and Sami, while the rest have limited or no knowledge of Sami (25,26). Four criteria determine bilingualism: (a) speaking the language of origin, (b) linguistic competence, (c) the function (use) of language and/or (d) attitude (identity and identification with language and/or culture) (27). The latter is important because it includes bilingualism as “cultural identity and identification” irrespective of reduced language competence, which is relevant for the predominantly Norwegian-speaking Sami. The linguistic competence and function criteria emphasize flexibility and a variety of language choice in different situations (28). Bilinguals may talk about mental distress in different languages, in different ways, with different people, within different spheres, such as (a) intimate sphere (family), (b) personal sphere (friends), (c) social sphere (colleagues and acquaintances), and (d) public sphere (public authorities including health services^8^ (28–30)). Clusters of interaction and communication situations may occur between Sami patients and their interlocutors^9^ (29), in this case meaning family/friends or therapists. A limited possibility to speak Sami in their interaction with health services may be a reason for Sami patients’ reduced satisfaction with health services (18,20).

Another assumed reason for reduced satisfaction with health services is that cultural differences between Sami patients and therapists lead to interaction and communication problems (31–34). The concept of culture is a wide and complex concept with hundreds of definitions (35). We have not restricted our theoretical frame to one specific definition; our understanding of culture is a complex, dynamic, socially acquired pattern of behaviours that *influences*, but *does not determine*, peoples’ mindsets and modes of living. Culture often serves as essentialized explanations for what people do and how they “are,” as if culture defines one specific phenomenon and delimits specific “cultural traits” as belonging to one group of people exclusively. However, culture does not *act* or *cause* anything in itself, nor is it a static set of characteristics describing or explaining what groups of people think or do.

Culture serves as a foundation for our understanding of and acting in the world and is shaped by individual experiences in a socially positioned, political and historical context, as well as being influenced by new generations, gender, educational level, economy and geographical location (1,36,37). Culture is reproduced through a process where individuals learn its norms and values from others. Cultural norms are, often unconscious, guidelines for behaviour, interaction and communication in a group, and when internalized by individuals, norms are taken for granted and appear as “the normal way” to behave (30,38,39). People are influenced by cultural norms in what they consider as acceptable or usual topics to talk about, in what way and with whom (29,30). Culture is relevant for health services because patients are influenced by culture in how they experience, handle and express their mental health problems, as well as their help-seeking behaviour and response to treatment interventions (2). Describing and working towards understanding patients’ cultural backgrounds may help to illuminate the health issues as people experience them, and to do so without essentializing culture. This study explores how patients^10^ experience and handle cultural norms in a mental health treatment setting.

The aim of this study is to explore the significance of language and cultural norms in communication about mental distress, as experienced by Sami patients receiving mental health services. We will discuss how these experiences might enhance our understanding of linguistic and cultural adaptation of health services.

## Method and material

### Design

This explorative study investigated the significance of language choice and cultural norms in Sami patients’ encounters with mental health services.

A qualitative method was chosen as most suitable for exploring issues of which we have limited knowledge and for gathering information as experienced and narrated by the individuals themselves (40,41).

### Data collection

Data were collected through individual interviews with Sami patients in outpatient mental health treatment. The semi-structured interview guide included the recording of demographic data^11^ and questions about the significance of language and culture for mental health problems, treatment and communication with the therapist/health services. The first author conducted the interviews, which lasted for 60–165 minutes each. The participants chose their treatment locations to be the place for the interviews.

The interview language was Norwegian, as proposed in the invitation letter, since the interviewer did not speak Sami sufficiently. The participants were encouraged to talk freely, draw on their own experiences and raise issues relevant to them. However, such free talk may have been limited because of the lack of a Sami-speaking interviewer. An interpreter was offered but rejected by the participants, as they felt their fluency in Norwegian to be adequate. A bilingual interviewer might have accessed more stories about other experiences. The interviews were audiotaped and transcribed.

### Recruitment procedure

The study aimed to include men and women over 18 years in ongoing outpatient treatment from Northern, Lule and Southern Sami regions. We requested 7 mental health clinics in Northern Norway for permission to recruit from their patients. These institutions were chosen because they serve patients from the Sami Language Administrative District. Three of the 7 outpatient clinics, all in the Northern Sami region, permitted recruitment of their patients.

Explicit Sami self-identification was an inclusion criterion, independent of mother tongue or Sami language skills. Recruitment was not restricted by diagnosis, treatment duration, previous treatment series or reason for seeking help.

Letters containing information about the study, including the interview topics and an invitation to participate, were available to all patients in the arrival area visiting the clinics, or were distributed by their therapist from February 2012 to February 2013. Persons interested in participating submitted the consent form and left their phone number or e-mail address, and the interviewer contacted them for an appointment.

### Sample

Five patients submitted the consent form and participated in interviews. One of these was excluded because of no explicit identification as Sami. Two women and 2 men, aged 21–50 years were included.

The participants were born and lived in small communities, three in majority Sami-speaking inland areas, and one in a predominantly Norwegian-speaking coastal area. One was married and had children, while the other three were unmarried without children. Three had previously received mental health treatment. Three participants were bilingual; two of these had Sami as mother tongue and Norwegian as second language, while one was bilingual from birth with one Sami-speaking and one Norwegian-speaking parent. One had Norwegian as mother tongue and spoke some Sami.

### Analysis

To identify elements of meaning from the texts, we conducted a thematic text analysis, using an inductive approach and systematic text reduction (41–43).

A stepwise analysis was performed:

1. Reading all the interview texts and identifying those parts related to the research questions.
2. Meaning units, that is, fragments of text containing information about the research question were identified. The meaning units were condensed and coded, thus reducing the amount of text without losing the meaning.
3. Related code groups were categorized into themes.^12^
4. Based on the code groups, short analytical texts were formulated, elaborating the understanding of the themes and reflecting the essence of meaning in the original text (43).

## Findings

The presentation of findings is based on the analysis of the texts together with selected quotations. Table I presents examples of the analysis. From the analysis, we identified two themes: (I) Language choice is influenced by language competence, to whom one talks and what one talks about and (II) Cultural norms influence what one talks about, in what way and to whom.

**Table I:** Examples of analysis with meaning units, codes, code groups and themes

Meaning units: citations from the interviews	Codes	Code groups	Themes
I: In your communication with the therapist, can you express yourself equally well in Norwegian as you do in Sami? I mean, do you have better words for feelings in Sami than in Norwegian? Pa4: What do you mean? I: Do the Sami words describe how you are doing in a better way? Pa4: No. We don't have words like that in Sami. So emotionally, I think I express myself better in Norwegian, describe the situation … Just now, I can't think of words to describe what I feel. In Sami. I: What do you think about that? Pa4: It was thought provoking, what you said. But of course, we do have words for some states of feelings, such as pain. Such simple words we do have, but not deep … emotional …. But wait, in fact, if I think about it, we do have that kind of word. But it's sort of not used, I don't think I've said that, ever, don't think so. So it came as a shock when you asked. I had to start thinking, although my mother tongue is Sami, it's maybe not used so often, but you have sometimes heard it. But it's not something you use every day. I think Sami society doesn't talk much about feelings and problems.	Emotionally I express myself better in Norwegian. I cannot think of words to describe what I feel, in Sami. We have simple words for feelings such as pain in Sami, but not for deep emotions. If I think about it, we have such words for emotions, but they are so rarely used you forget about them. Sami society does not talk about feelings and problems.	Express emotions more easily in Norwegian. Lack of Sami language competence for emotions. Sami society does not talk about feelings and problems.	Theme I: Language choice is influenced by language competence, to whom one talks and what one talks about. Theme II: Cultural norms influence what one talks about, in what way and to whom.

### Theme I

The participants’ choice of language in different situations was influenced by language competence. The bilingual participants claimed to speak Norwegian and Sami equally well, and stated that being unable to use Sami consistently would not pose a problem. Switching between Sami and Norwegian was a normal part of everyday life and a natural way to relate to the world. One participant expressed it like this:

My mother tongue is Sami, but for me it doesn't make any difference whether I speak Sami or Norwegian. I have no difficulty expressing myself in Norwegian, I must say I think equally clearly in both languages, I can find the right words and talk about things in the same way in Norwegian as in Sami.

The Norwegian-speaking participant, however, described her lack of competence in Sami as a loss. She said that her mother tongue should have been Sami instead of Norwegian, but because of the assimilation process, the Sami language was not spoken in her family. As a child, she had tried to learn Sami to be able to speak with her grandparents, but gave up because they got angry with her for speaking Sami to them. She said that “*the Sami way of thinking*” influenced the way she talked, and that language was a matter of identity. She felt that her lack of fluency in Sami prevented her from adequately expressing her sense of identity and state of health.

That Saminess is very strong inside me […] I feel that Norwegian, well it's not my language, and it feels very good to get into a Sami milieu where people speak Sami. It's very much … the way of thinking, the way of talking …. So it's not only people with another language who need an interpreter. So therapists have to understand that even if someone is only speaking in Norwegian, it might not be Norwegian, the thing is that you don't have the words you need.

Language choice also varied with the person with whom they spoke. If both/all spoke Sami, they also spoke Sami. If one non-Sami speaker was present, they spoke Norwegian, which everybody understood. In the intimate family sphere, some spoke Sami with some family members and Norwegian with others. Two participants had siblings with different mother tongues; some had spoken Sami and others Norwegian since childhood. However, in the non-intimate health care sphere, all usually spoke Norwegian with their therapists.

It doesn't matter which language we talk, I would really have preferred Sami in fact, but if that's not possible, I speak Norwegian. That's no problem. But when we just speak among ourselves, we Sami speakers, we very readily shift to Sami, as it's easier, more natural. The psychologist I go to now is the first person to ask whether I'd prefer to speak Sami or Norwegian. I told him it really didn't matter, but I also told him that I once had a Sami-speaking therapist and it felt easier because we spoke in Sami.

Finally, language choice varied with different topics, in this case emotions and reindeer herding. Some had difficulty in communicating in Sami about “sensitive” topics such as emotions and mental health problems. One expressed it like this:

Just now, I can't think of any words to describe how I feel, in Sami. […] So, emotionally, to explain my situation … I express myself better in Norwegian […]

Even with their closest relatives with whom they normally spoke Sami, some did not talk in Sami about all topics. One participant spoke Sami with her parents, except about feelings, as she explained:

When I try to explain to my mother how I feel, I can't quite manage to do it in Sami; I don't know how to say it in Sami. Sami is a rich language, but it's easier to talk in Norwegian about feelings. I also write my diary in Norwegian, it's easier.

This participant preferred to speak in Sami about reindeer herding. She described reindeer herding as a specific Sami mode of living, as only Sami are allowed to own reindeer and the Sami language dominates this aspect of Sami society. Her need to explain the context for her mental health problems in her reindeer herding life to the therapist was complicated by lack of language. She said:

Sometimes it would be better if my therapist was a Sami … I've had to spend some time explaining things about reindeer herding so that [the Norwegian therapist] could understand how it works, because I can't find the Norwegian words, those kinds of words don't exist in Norwegian.

### Theme II

The participants were influenced by cultural norms in what they considered acceptable or unacceptable to talk about. They described 3 topics as not usual or acceptable to talk about in the Sami community: emotions, mental health problems and physical/sexual abuse.

One participant was rather shocked to find that he had never talked about his feelings in Sami, when during the interview he was asked whether Sami words worked better to describe how he was doing. He said:

No. We don't have words like that in Sami. So emotionally, I think I express myself better in Norwegian, describe the situation … Just now, I can't think of words to describe what I feel. In Sami. […] of course, we do have words for some states of feelings, such as pain. Such simple words we do have, but not deep … emotional …. But wait, in fact, if I think about it, we do have that kind of word. But it's they're sort of not used, I don't think I've said that, ever, don't think so. So it came as a shock when you asked. I had to start thinking, although my mother tongue is Sami, [such words] are maybe not used so often, but you have sometimes heard it. But it's not something you use every day. I think Sami society doesn't talk much about feelings and problems.

Some participants said they did not talk about these topics because one is not supposed to have mental problems, and not complain about them if one does, as one participant put it:

For a reindeer herder it's almost shameful, you're not supposed to speak out loud about having mental problems, because you can't have them. “There are no mental problems in reindeer herding, it's just something people have invented to have something to complain about,” that's what people say about it.

Several of the participants had experienced violence and/or sexual abuse in different ways, as victims or as abusers, but they had not talked about it before they started therapy.

I suffered violence and abuse, but it wasn't something I thought about then. And we didn't talk about it either. My mom is a Sami and I've probably taken after her in a way … I haven't talked about it as an adult either.

The participants were also influenced by cultural norms in their way of talking. All participants had experienced communication problems with the health services and considered cultural differences between themselves and the therapists to be the reason. They described the “Sami way of communicating” as a norm as being less verbal, more indirect, using hints and body language and not complaining. This was in contrast to the straightforward/confrontational way health providers communicate. One said it like this:

And then there's the thing that we don't like to talk much about personal stuff. To keep things to yourself is typically Sami. I don't talk much, I've never been a person who said much, and when I need to react to something, I go off by myself. Because that's a typical Sami thing, you tend to spend a long time working through things, and you don't talk.

The participants said that the “Sami way of communicating” influenced the way a Sami expresses a need for help or support.

The Sami way to ask for help is by hinting … because you don't want to force your needs on anyone. The way to tell others you're having a hard time is very indirect, cautious, roundabout, maybe as a joke …

You don't say you're in a crisis … because people don't say that. Everyone understands … everyone understood … if you just said … a little … and then … you almost didn't need to say anything, and people understood … in your family … they can tell by looking at you … how you are. I think body language is widely used in the Sami culture. Health providers should know about the Sami way of telling, the way a Sami asks for help.

The findings indicate that the participants were highly motivated for treatment, and decided to change their “Sami way” of communicating to get help. As one put it:

We don't talk so much about feelings and problems in Sami society, but I decided I had to be open about this, try to get help because I couldn't take it anymore. “Why am I here, is this anything to live for?” – at the end it was sometimes kind of that I wanted to get away, to the hereafter. Then I decided this can't go on. I can't manage this on my own.

Another said it like this:

So I actually had to change … I'm no longer like my parents, I use so many fancy words. I've had to change to get ready for … to enable me to get some actual help from psychology, in fact. Health providers should know about that, how Sami express things.

The participants’ choice of whom they talked to about sensitive topics was partly also influenced by cultural norms. Initially, the participants described the topics as “what we don't talk about” or “what we keep in the family,” but eventually they said that they do talk about them but only with selected trusted persons in whom they could confide. Some talked about emotions and mental health problems in the nuclear family but not in the extended family.

You can't talk about mental health problems in the reindeer herding society […] but as for me and my mom, we can talk about what we talked about [in treatment] and dad can also ask: Well, how did it go? But it's not like we go and tell others in the “siida.”^13^

Others had family members who did not accept communication about these topics and expected them to deal with the problems by themselves, expressed like this:

I told my mother I went to see a psychologist, but she just said, “What? A shrink? Can't you even cope with that thing?” We never talked about that again.

Some did not talk with anyone in the family but had one or two close friends as interlocutors.

However, when the mental problems had become so severe that the participants had difficulties functioning in daily life, for example, insomnia, feeling isolated, depressed or suicidal, they said communication only with family and friends was insufficient. The only option was to find someone outside to talk to, and they turned to the health care system.

[…] to come here [to the therapist], that's what was needed, I had no choice. You think you can deal with it on your own, but you can't, you need help. My God, where would I be if I hadn't gotten help? It was like my life was nothing, I was playing with my life.

Some preferred a local therapist who, sharing their cultural and/or ethnic background, was assumed to understand them better.

Because I come from the Sami culture I have trouble communicating with doctors from the south. They don't understand the nuances unless they happen to have that kind of personality that makes them realize …. I haven't met many who come from other places who understand. When I went to a Sami therapist, it worked well; there wasn't much misunderstanding between us.

Others preferred to talk to a non-local therapist as they felt they could talk more freely with someone they were unlikely to encounter in a private context. They found it unpleasant that relatives and acquaintances worked in the institution where they received treatment. They did not want people they knew to learn about their mental problems, because as previously mentioned, one is not supposed to have mental problems in the Sami society. Although they had faith in the therapists’ confidentiality, they were afraid of being thought of negatively or perhaps as crazy. One said it like this:

Emotionally it would have been easier to talk to someone I don't know in private. I think it would have been easier if I'd lived in a city, where you don't meet them [therapists] in the street or anywhere. My therapist is leaving, and when he leaves, there are only people I know left working as therapists, that's a crisis for me.

## Discussion

Sami language and culture are described as critical factors in health care for the Sami population, especially mental health (6,23,44,45). This has brought about legislation that entitles the Sami to use their language and receive culturally adapted health care services. This study shows that the issue of linguistic and cultural adaption of health services is more complex than the question of health policies and a legal right to receive adapted services. Sami patients are not one cultural group with common needs, rather they have individual preferences concerning choice of language and communication partners as well as how they view and handle cultural norms.

According to Helander (46), several factors influence language choice: whether the discourse situation is public or private, whether the role relationship between the interlocutors is intimate or not and whether the interlocutors are Sami or not. Helander (46) found coherence between language, relationships and spheres, in that Sami (intra-ethnic) interlocutors in intimate relationships in the private sphere spoke Sami, while inter-ethnic interlocutors in non-intimate relationships in the public sphere spoke the majority language.

The present study indicates that language choice does not necessarily follow relationships and spheres, but varies within both the private and public sphere, as well as in intimate and non-intimate relationships. Our participants interact and communicate in or across spheres, in one or the other language. Within intimate relations in the private sphere, members of a nuclear family do not necessarily share one common language. A person might speak Sami with one sibling or parent and Norwegian with the other, depending on each person's language competence. However, as in Helander's study (46), our findings show that the majority language, Norwegian, is the most common language in encounters with the health services, and most participants say they accept and handle that without problems. The one person who expresses language problems is actually the predominantly Norwegian-speaking participant. Her reflections about her lack of the right language to express the sense of Saminess inside her emphasize how important it is to explore patients’ linguistic and cultural background. A large proportion of the Sami population are Norwegian speakers but may still experience a lack of the Sami language to express their sense of cultural identification with language and/or culture (27). This suggests the possibilities of exploring non-verbal experiences and raises the question of whether the treatment language influences the treatment result, which should be further investigated.

Our findings suggest that language choice also depends on the topic and cultural norms that guide how and with whom certain topics are appropriate to discuss. Language choice is influenced by what Fishman (29) calls “topical regulation,” which implies that some topics are better handled in one language than the other. The variety of topics may lead bilingual speakers to acquire the habit of speaking about a certain topic in one language, because the other language or the individuals lack specialized terms or because it is considered strange or inappropriate to talk about it in the other language (29). In our study, the lack of specialized terms in Norwegian about reindeer herding hindered a satisfactory conversation about it with a Norwegian-speaking therapist. The lack of knowledge on specialized terms for emotions in Sami influenced some participants to use Norwegian with their therapists, but also with their closest relatives with whom they usually spoke Sami. Within a private sphere and in reindeer herding societies, the large number of Sami speakers would be expected to favor the use of Sami (46). However, a lack of terms or habit to talk about emotions influenced them to talk about it in Norwegian both in their families and in Sami society.

Pedersen (47) describes a similar finding: Sami participants attending an anxiety group said they lacked Sami terms about anxiety and were not habituated to talking about such issues. However, given this opportunity to talk about it, they managed to help each other to find the right Sami words to describe their emotions and anxiety problems. Historically, the Sami have had limited opportunity to develop terminology related to mental health because of the lack of a Sami-speaking health service, and according to our results, also because of the absence of the topic in Sami society and mass media and the influence of cultural norms (47).

People are influenced by cultural norms in what they consider acceptable and especially *unacceptable* actions and topics for conversation (39). Several authors refer to Sami norms influencing people to hide mental distress, manage on their own, not show weakness, keep problems such as depression to themselves or within the family and avoid seeking help (31,47–50). Culture is often used to explain human actions, assuming that people act as they do because of their culture. In such a deterministic perspective, culture appears as personal characteristics and cultural norms appear as “rules”, applicable to all Sami people (51). Discussing norms in a deterministic, generalized way involves the risk of creating essentialized myths about populations, such as “Sami do not talk about emotions.” However, norms are not static and unalterable, rather as culture they are relational, social constructions in continuous change. Our participants stated that they were aware of, but had chosen to bypass, the cultural norms of not talking about emotions and mental distress by doing just that – starting to talk about them. They described “the Sami way” of talking as using indirect communication, body language or hints and a lack of habit to talk about mental distress within Sami society. Our participants also changed their way of communicating to be able to receive the therapists’ treatment. They said that the open, public way of discussing mental health in Norwegian society enhanced their linguistic competence and possibilities to communicate their mental distress, in Norwegian. They could not cope alone and therefore sought help from the health services, which for them represented an arena for open-hearted communication about topics regulated by Sami norms. Our findings raise questions as to how health services should deal with such cultural nuances and balance respect for cultural norms with appropriate individual health care.

A study of bilingual public services in Sami-language administrative areas concluded that health services is the sphere where most people prefer to speak Sami but where the possibility is the poorest, because health providers are mostly monolingual Norwegian speakers (44). Norway has 2 official languages, Norwegian and Sami, but very few Norwegians have learnt to speak Sami (52). The limited number of Sami-speaking therapists reduces the possibility for Sami patients to choose one and speak Sami in their therapy.

In addition, even if therapists speak Sami, another challenge arises in the relationship to therapists as fellow villagers. The institutions in this study are located in small communities in Sami areas. Professional work in such communities raises challenges of proximity and distance between therapists and patients. Holding local and cultural knowledge could be an advantage for the therapist but might also imply a lack of necessary distance. Patients might have multiple social relationships with therapists as kinsmen, neighbours or acquaintances in the private spheres (53). A relative, neighbour or acquaintance will, as a health provider, access sensitive information that he/she might not have accessed through their private relationship. Some of our participants preferred to see a local and/or Sami therapist, assumed to have knowledge about Sami culture and the Sami way of communicating, while others saw this as raising dilemmas that overshadowed the language barrier. Most of these participants preferred a professional and non-intimate relationship to a therapist, where they meet exclusively in the public sphere; this was thus more important than the possibility to speak Sami with a local therapist.

Helander's study from 1984 revealed a tendency to use the majority and minority languages together in many situations. Now, 30 years later, we find the same tendency. Living as bilingual implies that language choice in different communication situations is influenced by a complex combination of various social and cultural factors (46). Our participants’ experiences reveal the importance of health care professionals learning about indigenous peoples’ history, politics, language and culture but ensures the individual perspective (54). Different patients have individual needs, varying opinions about and preferences for what they can talk about, in which language, in what way and with whom. The challenge for the health services is to allow for and safeguard such individual variations within the cultural framework of the patients.

## Limitations

The number of participants in this study was small and our findings are not valid for the entire Sami population. All participants were born and lived in the Northern Sami area, but other Sami, for example, from Lule or Southern Sami populations might have other experiences due to demographic, linguistic, individual and contextual differences, as well as differences in health services. The sample is especially limited in age, marital status and parenthood and these factors have not been considered, nor have other factors such as gender, class, education, occupation, living in rural/urban or Sami- or Norwegian-dominated areas. Finally, only participants attending therapy were included in this study. Persons whose treatment had terminated or those with no experience of treatment may have other issues, reflections and priorities associated with mental health services. A different demographic sample might have resulted in different findings.

The study was conducted in Norwegian. As the results show, the participants were fluent in Norwegian but expressed some things in Norwegian and others in Sami. A Sami-speaking interviewer could have explored and discussed Sami expressions in more detail with the Sami-speaking participants.

A broader sample and interviews in both Sami and Norwegian might reveal a broader range of meaning units associated with the significance of language and culture in mental health topics. An exploration of the participants’ experiences and reflections in both languages, and in both cultural perspectives, might have led to more or different findings.

## Concluding remarks

The present study indicates a complex situation where Sami patients’ choice of language and ways of communicating with mental health services are influenced by their language competence, cultural norms, the topic of conversation and their relationship with the interlocutors.

Most participants in this study perceive themselves as bilinguals, speaking Sami and Norwegian equally well. They consider bilingualism to be a resource and a natural part of their lives. Bilingualism enables flexibility and choice of language. There is a tendency to perceive the Sami as synonymous with Sami-speaking people (6,55), but many Sami people speak only or predominantly Norwegian. The participants’ language competence is even more complex; some might speak Norwegian fluently but still lack specialized terms for certain topics, such as reindeer herding, in Norwegian. Others, with Sami as their primary mother tongue, might still lack Sami terminology for mental distress and emotions. Individuals may be influenced by Sami cultural norms, which prevent a habit of talking about emotions and mental health issues, in families or in broader Sami society, inducing silence around such topics. Some Sami patients choose to bypass certain norms if they appear as obstacles to their well-being. Finally, language choice and the extent to which they speak about sensitive topics such as mental health also depend on the person with whom they speak. Some prefer a local/Sami and/or Sami-speaking therapist because of an assumed cultural competence. Others prefer a non-local therapist to keep professional distance, even though that means speaking Norwegian during therapy.

The discourse of cultural adaptation of health services to indigenous people is highly politicized, tending to essentialize the culture. Indigenous patients are often assumed to share certain cultural norms, values and beliefs to which health services have to adjust (2). Our study indicates a variety of language use and cultural influence. Patients are influenced by both Sami and Norwegian culture in their interaction and communication with health services. Bilingualism and knowledge of both Sami and Norwegian culture provide latitude and enhanced possibilities for both patients and the health services.

## Clinical implications

Access to useful mental health services for the Sami population may expand if patients can choose or switch between Sami or Norwegian language in therapy. They should also be able to choose between a local or non-local therapist. It is therefore important that health care facilities as well as health educational institutions recruit Sami-speaking health providers and students. Therapists and students should hold linguistic and analytical cultural competence and explore bilingual experiences and the diversity of cultural influence in handling and expressing mental health problems, without essentializing culture and stereotyping Sami patients.

## Further research

The number of participants in this study is small and should be followed up with a broader demographic sample to portray experiences of the Sami population in Norway as well as in other nations.

Sami-speaking researchers should conduct further research to explore Sami-speaking patients’ language competence and preference in communication about mental health topics. The meaning of language and culture in relation to professional contact about mental health issues in monolingual Sami-speaking populations, and in the Lule and Southern Sami populations where the minority–majority situation is different will probably add important understanding to the topic. It is important to include both individual and group variations when changes in health service programmes are to be discussed to prevent actions based on incomplete or biased knowledge.

This study has focused on Sami patients’ experiences, and further research should include exploration of the significance of therapists’ linguistic and cultural background and competence.

The focus and findings in this study are transferable, but must be contextualized, when exploring experiences of indigenous people in other countries.

## Ethics

The study was approved by the The Regional Ethical Committee (REK-number delivers on demand) and was conducted in accordance with the Helsinki Declaration of 1975, revised in 2008.

## Footnotes

1 Health status is outside the scope of this article and will not be further discussed.

## Footnotes

2 This study is limited to Sami patients in Norway.

## Footnotes

3 There are no census data on the number of Sami inhabitants because it is prohibited to register individual ethnicity data in Norway.

## Footnotes

4 The Patient Rights Act, § 3–5 regulates all patients’ right to information, adapted to individual qualifications, age, maturity, experience and cultural and linguistic background.

## Footnotes

5 The ILO Convention, Article 25 ensures the Sami people's right to be involved in formulating relevant health services and reaffirms Sami users’ right to adequate health care.

## Footnotes

6 In addition, SANKS and Finnmark Hospital Trust have made agreements to serve the Sami population in the counties of Lapland in Finland and Jämtland in Sweden.

## Footnotes

7 Health institutions are not obliged to include Sami-speaking staff even though Sami patients have the right to speak Sami; it is sufficient to offer interpretation service.

## Footnotes

8 For our purpose, we find it useful to define (a), (b) and (c) as “private/intimate,” in contrast to health services in the public/non-intimate sphere.

## Footnotes

9 Communication partners.

## Footnotes

10 In this study, we focus on the experiences of patients, not therapists, but we do realize that culture influences health providers to the same extent and should be equally examined.

## Footnotes

11 Age, occupation, mother tongue, Sami language competence, cultural/family background.

## Footnotes

12 Malterud calls this “category and sub-category headings”; here, we use the term theme.

## Footnotes

13 Reindeer herding society, usually including members of the extended family.

## References

[1] Browne AJ, Varcoe C, Jarvis C (2009). Cultural and social considerations in health assessment. Health assessment 1st Canadian edition.

[2] Kirmayer LJ (2012). Rethinking cultural competence. Transcult Psychiatry.

[3] King M, Smith A, Gracey M (2009). Indigenous health part 2: the underlying causes of the health gap. Lancet.

[4] Westerman T (2004). Guest editorial. Engagement of indigenous clients in mental health services: what role do cultural differences play?. AeJAMH.

[5] Gracey M, King M (2009). Indigenous health, part1: determinants and disease patterns. Lancet.

[6] Ministry of Health and Social Affairs (1995). Plan for helse- og sosialtjenester til den samiske befolkning i Norge, NOU 1995:6 (Plan for health and social services to the Sami Population in Norway. Norwegian Official Report NOU 1995).

[7] Kvernmo S (1997). Developing Sami health services – a medical and cultural challenge. Sami culture in a new era: the Norwegian Sami experience.

[8] Andresen A (2008). Health citizenship and/as Sami citizenship: Norway 1985–2007.

[9] Torgersen J (1956). Noen populasjonsgenetiske data fra samene (Some population genetical data from the Sami). Tidsskrift for den norske Lægeforening [J Norweg Med Assoc].

[10] Jonassen Ø (1959). Sosiale og hygieniske forhold i flyttsamenes basisområde (Social and hygienic conditions in basic nomad Sami area). Tidsskrift for den norske Lægeforening [J Norweg Med Assoc].

[11] Ryymin T (2008). Forebygging av tuberkulose i Finnmark 1900–60 (Prevention of tuberculosis in Finnmark county 1900–60). Tidsskrift for den norske Lægeforening [J Norweg Med Assoc].

[12] Sjölander P (2011). What is known about the health and living conditions of the indigenous people of northern Scandinavia, the Sami?. Glob Health Action.

[13] Hassler S, Kvernmo S, Kozlov A, Young TK, Bjerregaard P (2008). Sami. Health transitions in arctic populations.

[14] Kvernmo S (2004). Mental health of Sami youth. Int J Circumpolar Health.

[15] Turi AL, Bals M, Skre I, Kvernmo S (2009). Health services use in indigenous Sami and non-indigenous youth in North Norway: a population based survey. BMC Public Health.

[16] Gaski M (2011). Aspects of health services in Sami areas.

[17] Hansen KL (2011). Ethnic discrimination and bullying in relation to self-reported physical and mental health in Sami settlement areas in Norway: the Saminor study.

[18] Nystad T, Melhus M, Lund E (2008). Sami speakers are less satisfied with general practitioners’ services. Int J Circumpolar Health.

[19] Møllersen S (2007). Mental health services and treatment in Sami and non-Samipopulations: a comparative study in a multiethnic rural area of North Norway.

[20] Sørlie T, Nergård JI (2005). Treatment satisfaction and recovery in Sami and Norwegian patients following psychiatric hospital treatment: a comparative study. Transcult Psychiatry.

[21] Sami Parliament Helse og sosial (Health and social issues). http://www.sametinget.no/Helse-og-sosial.

[22] Minde H (2005). Assimilation of the Sami – implementation and consequences. J Indigen Peoples Rights.

[23] Ministry of Labour and Social Affairs (2008). St.meld.; nr. 28 (2007–2008); Samepolitikken (White paper; The Sami policy).

[24] Vangsnes ØA, Apneseth O, Botheim GS, Vangsnes ØA, Stibbe S (2013). To språk gir meir enn berre to språk (Two languages adds more than only two languages). Det Fleirspråklege Noreg (The multilingual Norway).

[25] Todal J, Todal J, Brustad M, Broderstad EG, Johansen K, Severeide PI (2013). Kvantitative endringar i den samiske språksituasjonen i Noreg. Ei kunnskapsoppsummering (Quantitative changes in the Sami language situation in Norway. A review).

[26] Helander N, Bull T, Lindgren AR (2009). Samisk språk og samiske språkforhold: med særlig vekt på nordsamisk (Sami language and language affairs, emphasizing Northern Sami). De mange språk i Norge Flerspråklighet på norsk [The many languages in Norway multilingualism in Norwegian our translation].

[27] Skutnabb-Kangas T (1981). Tvåspråkighet (Bilingualism).

[28] Hyltenstam K, Stroud C (1991). Språkbyte och språkbevarande: om samiskan och andra minoritetsspråk (Language change and language maintenance: about Sami and other minority languages).

[29] Fishman JA (1965). Who speaks what language to whom and when?. La Linguistique.

[30] Dahl Ø (2001). Møter mellom mennesker: interkulturell kommunikasjon (Encounters between human beings: intercultural communication).

[31] Bongo BA (2012). “Samer snakker ikke om helse og sykdom”: samisk forståelseshorisont og kommunikasjon om helse og sykdom: en kvalitativ undersøkelse i samisk kultur (Sami don't talk about health and illness. Sami horizon of understanding and communication about health and illness.

[32] Hedlund M, Moe A (2000). “De forstår ikke hva som er viktig for oss”: helsetjenester og sørsamer (“They don't understand what's important to us”: health services and South Sami).

[33] Kuperus K (2001). Kartlegging av behovet for tilrettelagte helse- og sosialtjenester for det samiske folk på Helgeland (Mapping of the need for personalized health and social services for the Sami people in Helgeland).

[34] Olsen T, Eide AK (1999). “Med ei klype salt”: håndtering av helse og identitet i en flerkulturell sammenheng (“With a pinch of salt”: dealing with health and identity in a multicultural context).

[35] Kroeber AL, Kluckhohn C (1952). Culture: a critical review of concepts and definitions.

[36] Barth F, Vermeulen H, Govers C, Barth F (1994). Enduring and emerging issues in the analysis of ethnicity.

[37] Wikan U, Evans TD, Frønes I, Kjølsrød L (1994). Folk- ikke kulturer – kan møtes (People – not cultures – can meet). Velferdssamfunnets barn (The children of the welfare society).

[38] Banwell C, Ulijaszek SJ, Dixon J (2013). Introduction, in when culture impacts health: global lessons for effective health research.

[39] Eriksen TH (1998). Små steder – store spørsmål: innføring i sosialantropologi (Small places, large issues: an introduction to social and cultural anthropology).

[40] Creswell JW (2013). Qualitative inquiry & research design. Choosing among five approaches.

[41] Malterud K (2011). Kvalitative metoder i medisinsk forskning: en innføring (Qualitative methods in medical research: an introduction).

[42] Malterud K (2001). Qualitative research: standards, challenges, and guidelines. Lancet.

[43] Malterud K (2012). Systematic text condensation: a strategy for qualitative analysis. Scand J Public Health.

[44] Skålnes S, Gaski M (2000). Tospråklig tjenesteyting: brukerundersøkelse i forvaltningsområdet for Samelovens språkregler (Bilingual services: consumer survey in Sami language administration areas).

[45] Solstad KJ, Balto ÁMV (2012). Samisk språkundersøkelse 2012 (Sami language investigation 2012).

[46] Helander E (1984). Om trespråkighet: en undersökning av språkvalet hos samerna i Övre Soppero (About trilingualism: a study of language choice among the Sami in Upper Soppero). [Doctoral Thesis].

[47] Pedersen J, Silviken A, Stordahl V (2010). Erfaringer fra kurs i angstmestring for samisktalende (Experiences from a course in anxiety coping for Sami speakers). Samisk psykisk helsevern: nye landskap, kjente steder og skjulte utfordringer (Sami mental health: new landscapes, familiar places and hidden challenges).

[48] Gerhardsen E, Silviken A, Stordahl V (2010). “Jeg skjønner meg ikke på dem”: om kulturelle faktorer i kommunikasjon (I don't understand them: on cultural factors in communication).

[49] Kaiser N, Ruong T, Renberg ES (2013). Experiences of being a young male Sami reindeer herder: a qualitative study in perspective of mental health. Int J Circumpolar Health.

[50] Silviken A, Berntsen G, Dyregrov K (2014). Etterlattes erfaringer med lokalt hjelpeapparat i samiske områder i Nord-Norge (Bereaveds’ experiences with local support system in Sami areas in northern Norway). Sykepleien Forskning [Nurs Res].

[51] Sørensen BW (1997). Når kulturen går i kroppen. ‘Halve grønlaendere’ som begreb og faenomen (When culture gets into the body. “Half Greenlanders” as a concept and a phenomenon). Tidsskriftet Antropologi.

[52] Helander N, Mørck E, Magga T (2002). Flerspråklighet i det samiske samfunnet (Bilingualism in the Sami society). Samiska i et nytt årtusende (Sami in a new millenium).

[53] Kemi RR, Eidheim H, Stordahl V (1998). Om å være profesjonell i lokalsamfunn (To be professional in a local community).

[54] Blix BH (2014). A critical reflection on the importance of culture between immigrants and health care services. Tidsskrift for samfunnsforskning [J Soc Res].

[55] Blix BH, Hamran T, Normann HK (2013). “The old Sami” – who is he and how should he be cared for? A discourse analysis of Norwegian policy documents regarding care services for elderly Sami. Acta Borealia.

